# Covid-19 vaccination and menstrual bleeding disturbances among women of fertile age: a Norwegian registry study

**DOI:** 10.1007/s10654-024-01170-0

**Published:** 2024-11-06

**Authors:** Maria C. Magnus, Ida H. Caspersen, Knut-Arne Wensaas, Helena N. Eide, Anne K. Örtqvist, Laura Oakley, Per Magnus, Siri E. Håberg

**Affiliations:** 1 Centre for Fertility and Health, Norwegian Institute of Public Health, P.O. Box 222, Skøyen, Oslo, 0213 Norway; 2 Research Unit for General Practice, NORCE Norwegian Research Centre, Bergen, Norway; 3 Institute of Health and Society, University of Oslo, Oslo, Norway; 4 Clinical Epidemiology Division, Department of Medicine, Karolinska Institutet, Solna, Stockholm, Sweden; 5 Department of Obstetrics and Gynecology, Visby County Hospital, Visby, Sweden; 6 Department of Non-communicable Disease Epidemiology, London School of Hygiene and Tropical Medicine, London, UK; 7 Department of Global Public Health and Primary Care, University of Bergen, Bergen, Norway

**Keywords:** Covid-19 vaccination, Menstrual bleeding disturbances

## Abstract

This study evaluated the relationship between Covid-19 vaccination and menstrual bleeding disturbances using a large national registry linkage including 666,467 women between 20 and 40 years of age residing in Norway on January 1st, 2019. Information on vaccination—BNT162b2 and mRNA-1273 — was obtained from the Norwegian vaccination registry. Diagnoses of menstrual disturbances (absent/scanty, excessive, irregular/frequent menstruation, and intermenstrual bleeding) was obtained from the general practitioner database. We examined new-onset menstrual bleeding disturbances using a Cox regression comparing vaccinated to unvaccinated women, where women contributed follow-up time as unvaccinated until the day of vaccination. In addition, we conducted a self-controlled case-series analysis, and a sensitivity analysis excluding all those who remained unvaccinated throughout the pandemic, to evaluate the role of unmeasured confounding. We observed an increased risk of several menstrual bleeding disturbances after vaccination against Covid-19, ranging from an adjusted HR (aHR) of 1.18 (95% CI: 1.04, 1.33) for intermenstrual bleeding to 1.29 (95% CI: 1.23, 1.36) for irregular/frequent menstrual periods. However, estimates were fully attenuated when excluding women who remained unvaccinated at the end of follow-up (aHRs between 0.97 and 1.08). No differences were identified according to vaccine dose or type. Our self-controlled case series analysis confirmed no increased risk after a first dose of vaccination, though there was a slightly increased risk of menstrual bleeding disturbances from 61 days after vaccination with dose 2. In conclusion, the modestly increased risk of menstrual bleeding disturbances after Covid-19 vaccination appeared to reflect a role of unmeasured confounding by women who never received Covid-19 vaccinations, as associations did not remain when risk after vaccination were compared to risk before vaccination among ever vaccinated women.

**Supplementary Information:**

The online version contains supplementary material available at 10.1007/s10654-024-01170-0.

## Introduction

Among women of reproductive age, menstrual bleeding disturbances are common, estimated to affect approximately one in three women, and include for example absent menstruation, heavy menstrual bleeding, irregular periods and intermenstrual bleeding [1, 2]. Recent meta-analyses summarizing the current evidence support a transient increase in the cycle length and heavier menstrual bleeding (greater menstrual flow) after vaccination against Covid-19 [3, 4]. However, the vast majority of existing studies were at moderate-to-high risk of bias due to a retrospective design, interviewer bias, and failure to include a non-vaccinated control group [3, 4]. Despite this, due to the existing evidence at the time, the European Medicines Agency recommended that heavy menstrual bleeding be included as a potential side effect of the Covid-19 mRNA vaccines in October 2022, whilst they stated that there was insufficient evidence to indicate that absence of menstruation was a potential side effect of the vaccines [5].

The largest prospective study available, a registry-based study from Sweden, attempted to mitigate limitations of existing studies by using population-based registries to compare prospective registrations of menstrual disturbances between unvaccinated and vaccinated individuals [6], providing no support for a causal association between Covid-19 vaccination and menstrual bleeding disorders. Notably, this Swedish study was primarily based on registrations of menstrual disturbances in specialist health-care services, thereby not being able to fully capture menstrual disturbances only seen by general practitioners and did not evaluate specific types of menstrual disturbances. Additional studies which do not select participants based on outcome status, with prospectively collected data on both vaccination and menstrual characteristics, with an unvaccinated comparison group, and the possibility to look at a broader range of menstrual bleeding disturbances, are therefore warranted.

Covid-19 vaccines have been linked to various coagulation and bleeding disorders [7–9], potentially explained by a combined effect of the SARS-CoV-2 spike protein and adenovirus vector-triggered signalling pathways, supporting a role of Covid-19 vaccination in bleeding disturbances particularly after the adenovirus vector-vaccines. Less is known about how the mRNA vaccines might cause bleeding disturbances. A biological explanation for how vaccination might influence menstrual bleeding disturbances remains elusive. A role of estrogen receptors on immune function is documented [10, 11], and while a potential influence of the immune system on estrogen levels is unclear, it seems plausible that both stress and inflammation could impact ovarian hormones and menstrual bleedings [12].

The objective of the current study was therefore to evaluate the relationship between Covid-19 vaccination with the mRNA vaccines and risk of new-onset menstrual bleeding disturbances using a Norwegian registry linkage with health care data on all women in Norway ages 20–40. We hypothesized that women would experience transient menstrual bleeding disturbances after Covid-19 vaccination.

## Materials and methods

### Study population

We studied all women between 20 and 40 years of age residing in Norway on January 1st, 2019. We excluded teenagers and those aged over 40 (as a proxy for peri- and post-menopausal women) as the timing of menarche and menopause is not recorded in the registries. Data in this study were provided through the Emergency Preparedness Register for Covid-19 (Beredt C19), administered by the Norwegian Institute of Public Health, according to the Health Preparedness Act § 2–4 [13]. Information on registered individuals was obtained from the Norwegian Immunization Registry (SYSVAK), the Norwegian Surveillance System for Communicable Diseases (MSIS), the Medical Birth Registry of Norway (MBRN), the Patient Registry (NPR), the Norwegian Control and Payment of Health Reimbursements Database (KUHR; includes all registrations in primary care) and Statistics Norway (SSB). Information from all data sources were linked using unique national identification numbers assigned to all individuals residing in Norway at birth or immigration. Additional information on the various registries is available in the supplement methods. We excluded women who did not have a valid national identification number, women who had received other Covid-19 vaccines than the mRNA vaccines from Pfizer-BioNTech [BNT162b2] and Moderna [mRNA-1273], and women with a history of any menstrual bleeding disturbances during the two years prior to the start of follow-up (from January 1^st^, 2017).

### Covid-19 vaccination

We obtained information on the dates and types of all Covid-19 vaccine doses from SYSVAK, which includes mandatory registrations of absolutely all Covid-19 vaccines administered in Norway. The two mRNA vaccines from Pfizer-BioNTech (BNT162b2) and Moderna (mRNA-1273) and one viral vector vaccine from AstraZeneca (AZD1222) were part of the national vaccination program, although the AZD1222 vaccine was excluded from the vaccination program on May 12, 2021, and after this only the two mRNA vaccines were available in the program. In Norway, women with risk factors for severe disease or for being exposed through their work in the healthcare sector were gradually offered the vaccine from January 2021, while everyone over 18 years had been offered their first dose by the end of August 2021. For the purpose of this study, we first looked at having received at least one dose of one of the two mRNA Covid-19 vaccines (yes vs. no as time-varying exposure), and subsequently evaluated the association with the number of vaccine doses (none, 1, 2 and 3 or more doses as time varying exposures), and finally examined the association with the two mRNA vaccines separately (only BNT162b2, only mRNA-1273, and mixed, as compared to those unvaccinated). We also evaluated differences according to time since the first vaccine dose (0–60 days, 61–120 days, 121–180 days, and more than 180 days). The Norwegian government recommended a minimum of three weeks between the first two doses, and a minimum of four months between dose 2 and 3 [14].

### Menstrual bleeding disturbances

We identified diagnoses of menstrual bleeding disturbances made by general practitioners in the KUHR-database. Diagnoses and symptoms in primary care are made according to the International Classification of Primary Care version 2 (ICPC-2). In this study, we evaluated the codes for “menstruation absent/scanty” (X05), “menstruation excessive” (X06), “menstruation irregular/frequent” (X07), and “intermenstrual bleeding” (X08). We first evaluated any registration of any of these menstrual bleeding disturbances, and subsequently these outcomes were all evaluated separately.

### Covariates

We also obtained information on age at start of follow-up, household income (categorized according to tertiles), educational level (categorized as 9 years or less, 10–12 years, more than 12 years, and missing), marital status (single, married/registered partner, divorced/separated, other/unknown), and region of birth (Scandinavia, other European countries, middle East/Africa, other/unknown) from Statistics Norway. We further identified pre-existing registrations prior to the start of follow-up (yes vs. no) in the patient registry of endometriosis (International Classification of Diseases version 10 [ICD-10] code N80), polycystic ovarian syndrome (ICD-10 code E28.2), diabetes mellitus (ICD-10 codes E10 and E11) and thyroid disorders (ICD-10 codes E00-07). Information on all positive PCR-tests for SARS-CoV-2 was obtained from MSIS.

### Statistical analysis

We used a Cox proportional hazards regression to estimate risk of new-onset registration of any menstrual bleeding disturbance, excluding those with any registration of menstrual bleeding disturbances prior to the start of follow-up (going back to January 1^st^ 2017). Vaccination against Covid-19 was entered as a time-varying exposure. Women therefore contributed follow-up time as unvaccinated until they became vaccinated with their first dose. The start of follow-up was January 1^st^, 2019, while the end of follow-up was the first date of the menstrual disorder of interest, emigration, death, or May 5^th^, 2023. We did not permit women to have multiple registrations of the menstrual disturbance of interest, meaning that women were censored at their first registration. However, for the analyses of the individual menstrual disturbances, women experiencing other disturbances were permitted to be part of the reference group. All pregnant follow-up days were excluded from the analysis. This means that women did not contribute with follow-up time from the start (birth date minus gestational duration at delivery) until the end of pregnancy. All pregnancies, including miscarriages, induced abortions, stillbirths and live births were identified as previously described [15]. Women who were currently pregnant were identified using antenatal care codes in both primary and secondary care, as previously described [16], and we excluded follow-up time starting from eight weeks before the first antenatal visit for ongoing pregnancies. We evaluated any Covid-19 vaccination, the number of doses, and the vaccine subtypes as time-varying exposures. The multivariable models adjusted for age at start of follow-up, income, education, marital status, region of birth, underlying chronic conditions and confirmed Covid-19 (positive test for SARS-CoV-2; time-varying covariate). Missing data on educational level, income and region of birth was dealt with by including a separate missing category for these covariates, as we believe this mostly reflected a lack of records in the Norwegian system for immigrants. We did not conduct multiple imputations due to the modest amount of missing data and limited information available to inform an imputation model. A possible side effect of menstrual bleeding disturbances after Covid-19 vaccination was suspected by the Norwegian Medicines Agency end of June 2021 [5]. This news story was circulated in the Norwegian media over the following days. To evaluate the role of the increasing media attention on the potential association between Covid-19 vaccination and menstrual disturbances, we conducted a stratified analysis, comparing before and after July 1^st^, 2021. As women who were not vaccinated throughout the Covid-19 pandemic may have different unmeasured characteristics than those who followed vaccine recommendations, we also conducted a sensitivity analysis excluding women who remained unvaccinated at the end of follow-up (May 5^th^, 2023), which was around the time WHO declared the pandemic to have ended (June 2023) [17]. To investigate the role of infection with Covid-19 on the associations, we stratified the relationship between vaccination against Covid-19 and risk of menstrual bleeding disturbances according to whether the women had experienced an infection or not by the end of follow-up.

To further evaluate the role of unmeasured confounding, we also explored the association between vaccination against Covid-19 and risk of menstrual bleeding disturbances by using a self-controlled case series analysis [18]. This analytical design was first developed to investigate associations between vaccination and acute potential adverse events. It is by design restricted to exposed cases (e.g. women who have been vaccinated and also have a registration of a menstrual bleeding disturbance) and the woman acts as her own control in the analyses. In this design we therefore account for all measured and unmeasured characteristics within women that do not change during the follow-up time. An important assumption of this analysis is that the experience of one event, in our case the registration of one menstrual bleeding disturbance, does not increase the likelihood of experiencing another event. Furthermore, the model assumes that the experience of the outcome (e.g. menstrual bleeding disturbances) does not alter the risk of the subsequent exposure (e.g. vaccination or infection). We compared the likelihood of being diagnosed with a menstrual bleeding disturbances 60 days before dose 1, 60 days after dose 1, and 61 to 180 days after dose 1, with the risk observed during the 61 to 180 days prior to being given dose 1. The incidence rate ratios for each of the time windows was calculated compared to the rate during the 180 − 61 days before dose 1 was given. We also evaluated the risk according to days since dose 2 as a sensitivity analysis, as it is after this that individuals are fully immunized and therefore have a more notable immune response [19, 20], and because previous studies indicated some evidence of a potential increased risk of menstrual bleeding disturbances after dose 2 [21–23].

As a secondary analysis, we evaluated a potential impact of confirmed Covid-19 infection on menstrual bleeding disturbances. Information on at least one positive PCR-test for SARS-CoV-2, as registered in MSIS, was entered as a time-varying exposure in a Cox regression, and we subsequently evaluated the risk according to the amount of time since the infection. We also conducted a sensitivity analysis censoring the follow-up time on January 31st, 2022, because from this date the Norwegian government ceased recommending testing for individuals with symptoms or those who had been in contact with confirmed cases.

All analyses were conducted in Stata version 16 (Statacorp, Texas).

## Results

There were 707,456 women between 20 and 40 years of age registered as residing in Norway as of January 1st, 2019, with a valid national identification number. After excluding women who had received other Covid-19 vaccines than the two mRNA vaccines, this left 666,467 women available for analysis. The median number of days between dose 1 and 2 was 43 (IQR 35, 60), while the median number of days between dose 2 and 3 was 191 (IQR 161, 224). Older women, women with higher education, women in the highest income tertile, women born in Scandinavia, and women with diabetes, had a greater number of Covid-19 vaccine doses (Table 1). After excluding women with registrations of menstrual bleeding disturbances prior to the start of follow-up, the rate of new registrations of bleeding disturbances per 100,000 person days of follow-up was 6.2 for any menstrual bleeding disturbance, 1.2 for menstruation absent/scanty, 1.5 for menstruation excessive, 3.5 for irregular/frequent menstrual periods and 0.6 for intermenstrual bleeding.

**Table 1:** Distribution of background characteristics according to vaccination status

Background characteristics	All included individuals (*N* = 666,467)	One vaccine dose by the end of follow-up (*N* = 28,512)	Two vaccine doses by the end of follow-up (*N* = 368,715)	Three or more vaccine dose by the end of follow-up (*N* = 174,349)	Remained unvaccinated at the end of follow-up (*n* = 94,891)
**Age at start of follow-up**,** N (%)**					
20–24	156,029 (23.4)	7,383 (25.9)	88,392 (24.0)	40,772 (23.4)	19,482 (20.5)
25–29	175,396 (26.3)	7,902 (27.7)	97,920 (26.6)	42,032 (24.1)	27,542 (29.0)
30–34	172,167 (25.8)	7,288 (25.6)	94,599 (25.7)	43,457 (24.9)	26,823 (28.3)
35–40	162,875 (24.4)	5,939 (20.8)	87,804 (23.8)	48,088 (27.6)	21,044 (22.2)
**Educational level**,** N (%)**					
9 years or less	108,985 (16.4)	7,092 (24.9)	58,164 (15.8)	22,832 (13.1)	20,897 (22.0)
10–12 years	170,872 (25.6)	7,616 (26.7)	99,438 (27.0)	44,024 (25.3)	19,794 (20.9)
More than 12 years	335,364 (50.3)	10,638 (37.3)	194,264 (52.7)	102,456 (58.8)	28,006 (29.5)
Missing	51,246 (7.7)	3,166 (11.1)	16,849 (4.6)	5,037 (2.9)	26,194 (27.6)
**Houseshold income**,** N (%)**					
1st tertile	218,098 (32.7)	10,756 (37.7)	114,777 (31.1)	54,069 (31.0)	38,496 (40.6)
2nd tertile	215,609 (32.4)	9,870 (34.6)	123,015 (33.4)	54,621 (31.3)	28,103 (29.6)
3rd tertile	212,996 (32.0)	6,988 (24.5)	127,667 (34.6)	64,541 (37.0)	13,800 (14.5)
Missing	19,764 (3.0)	898 (3.2)	3,256 (0.9)	1,118 (0.6)	14,492 (15.3)
**Marital status**,** N (%)**					
Unmarried	409,122 (61.4)	16,746 (58.7)	226,840 (61.5)	108,614 (62.3)	56,922 (60.0)
Married/registered partner	213,555 (32.3)	9,475 (33.2)	118,993 (32.3)	55,666 (31.9)	30,421 (32.1)
Divorced/separated	42.790 (6.4)	2,291 (8.0)	22,882 (6.2)	10,069 (5.8)	7,548 (8.0)
**Region of birth**^**a**^, **N (%)**					
Scandinavia	500,278 (75.1)	17,259 (60.5)	294,527 (79.9)	145,781 (83.6)	42,711 (45.0)
Other European countries	77,756 (11.7)	4,805(16.9)	29,768 (8.1)	9,939 (5.7)	33,244 (35.0)
Middle/East Africa	33,304 (5.0)	4,087 (14.3)	16,791 (4.6)	3,644 (2.1)	8,782 (9.3)
Other countries	55,129 (8.3)	2,361 (8.3)	27,629 (7.5)	14,985 (8.6)	10,154 (10.7)
**Endometriosis**,** N (%)**					
No	662,677 (99.4)	28,370 (99.5)	366,646 (99.4)	173,263 (99.4)	94,398 (99.5)
Yes	3,790 (0.6)	142 (0.5)	2,069 (0.6)	1,086 (0.6)	493 (0.5)
**Polycystic ovarian syndrome**,** N (%)**					
No	664,290 (99.7)	28,418 (99.7)	367,617 (99.7)	173,610 (99.6)	94,645 (99.7)
Yes	2,177 (0.3)	94 (0.3)	1,098 (0.3)	739 (0.4)	246 (0.3)
**Thyroid disorder**,** N (%)**					
No	660,844 (99.2)	28,294 (99.2)	365,792 (99.2)	172,565 (99.0)	94,193 (99.3)
Yes	5,623 (0.8)	218 (0.8)	2,923 (0.8)	1,784 (0.1)	698 (0.7)
**Diabetes mellitus**,** N (%)**					
No	664,290 (99.7)	28,399 (99.6)	367,551 (99.7)	171,763 (98.5)	94,578 (99.7)
Yes	2l177 (0.3)	113 (0.4)	1,164 (0.3)	2,586 (1.5)	313 (0.3)
**Confirmed Covid-19 by end of follow-up**,** N (%)**					
No	442,721 (66.4)	11,664 (40.9)	228,569 (62.0)	140,093 (80.4)	62,395 (65.8)
Yes	223,746 (33.6)	16,848 (59.1)	140,146 (38.0)	34,256 (19.7)	32,496 (34.2)
**Prevalent menstrual bleeding disorder**,** N (%)**					
No	634,327 (95.2)	26,879 (94.3)	350,611 (95.1)	165,763 (95.1)	91,075 (96.0)
Yes	32,140 (4.8)	1,633 (5.7)	18,104 (4.9)	8,587 (4.9)	3,816 (4.0)

^**a**^ Other countries includes North America, South America, Latin America, Asia, Australia, and New Zealand

### Survival analysis of Covid-19 vaccination and risk of menstrual disturbances

We observed a modest increased risk of all the evaluated menstrual bleeding disturbances after vaccination against Covid-19, ranging from an adjusted HR (aHR) of 1.18 (95% CI: 1.04, 1.33) for intermenstrual bleeding to 1.29 (95% CI: 1.23, 1.36) for irregular/frequent menstrual periods (Table 2). However, when we excluded women who remained unvaccinated (94891/14%) at the end of follow-up, the results attenuated, and we no longer observed an increased risk of menstrual bleeding disorders, with aHRs ranging between 0.97 (95% CI: 0.88, 1.06) for absent/scanty menstruation and 1.08 (95% CI: 0.94, 1.23) for intermenstrual bleeding (Table 2). There was no notable differences according to the number of vaccine doses (Table 3), or between the two different mRNA vaccines evaluated (Supplementary Table S1). After dose 1, we observed an increased risk of absent/scanty menstrual periods starting from the first menstrual cycle after vaccination, while a higher risk of excessive and irregular/frequent menstrual bleeding was observed from 61 days onwards, and an increased risk of intermenstrual bleeding only observed after more than 180 days, although these estimates were also substantially attenuated after excluding women who remained unvaccinated at the end of follow-up (Table 4). In the analysis stratified before and after July 1st, 2021, we found some evidence of an increased risk after July 1st, 2021, only, but the confidence intervals were overlapping indicating no robust evidence of a difference according to calendar time (Supplementary Table S2). Stratifying the association between Covid-19 vaccination and menstrual bleeding disturbances according to whether the woman had experienced an infection with Covid-19 by the end of follow-up or not, indicated that the associations were of a greater magnitude among those who had never been infected, but similar to what was observed in the other analyses this was completely attenuated when excluding women who remained unvaccinated at the end of follow-up (Supplementary Table S3).

**Table 2:** Covid-19 vaccination and bleeding disturbances among women between 20 and 40 years of age

Outcome	Exposure group	Full sample				Excluding those who remain unvaccinated at the end of follow-up			
		Follow-up time in days	N Cases	Unadjusted HR (95% CI)	Adjusted HR (95% CI)	Follow-up time in days	N Cases	Unadjusted HR (95% CI)	Adjusted HR (95% CI)
Any menstrual bleeding disturbance	Unvaccinated	534,178,634	32,420	1.00	1.00	429,781,852	27,441	1.00	1.00
	Vaccinated	302,998,237	19,893	1.24 (1.19 to 1.29)	1.29 (1.24 to 1.34)	302,998,237	19,893	0.96 (0.90 to 1.03)	1.03 (0.96 to 1.10)
Menstruation absent/scanty	Unvaccinated	567,184,386	7111	1.00	1.00	640,228,524	7,824	1.00	1.00
	Vaccinated	334,155,299	3746	1.06 (0.97 to 1.15)	1.21 (1.11 to 1.32)	150,377,272	1,875	0.91 (0.83 to 1.00)	0.97 (0.88 to 1.06)
Menstruation excessive	Unvaccinated	567,122,438	7791	1.00	1.00	639,456,131	9,454	1.00	1.00
	Vaccinated	333,117,611	5414	1.17 (1.08 to 1.25)	1.26 (1.17 to 1.36)	150,008,599	2,412	0.95 (0.88 to 1.03)	0.99 (0.91 to 1.07)
Irregular/frequent menstrual periods	Unvaccinated	553,425,740	18,531	1.00	1.00	620,676,554	22,252	1.00	1.00
	Vaccinated	320,056,403	12,419	1.26 (1.20 to 1.33)	1.29 (1.23 to 1.36)	144,607,500	5,872	0.95 (0.90 to 1.00)	0.97 (0.92 to 1.03)
Intermenstrual bleeding	Unvaccinated	571,951,296	3230	1.00	1.00	646,276,829	3,759	1.00	1.00
	Vaccinated	338,244,469	1923	1.14 (1.01 to 1.29)	1.18 (1.04 to 1.33)	152,077,275	883	1.04 (0.92 to 1.19)	1.08 (0.94 to 1.23)

Adjusted for age at start of follow-up, income, education, marital status, region of birth, endometriosis, polycystic ovarian syndrome, diabetes mellitus, thyroid disorders and confirmed Covid-19

**Table 3:** Doses of Covid-19 vaccination and bleeding disturbances among women between 20 and 40 years of age

Outcome	Exposure group	Full sample				Excluding those who remain unvaccinated at the end of follow-up			
		Follow-up time in days	N Cases	Unadjusted HR (95% CI)	Adjusted HR (95% CI)	Follow-up time in days	N Cases	Unadjusted HR (95% CI)	Adjusted HR (95% CI)
Any menstrual bleeding disturbance	Unvaccinated	534,178,634	32,420	1.00	1.00	429,781,852	27,441	1.00	1.00
	Dose 1	35,197,851	2250	1.26 (1.19 to 1.33)	1.23 (1.17 to 1.30)	35,197,851	2250	0.99 (0.92 to 1.07)	1.01 (0.94 to 1.09)
	Dose 2	205,288,899	13,768	1.24 (1.19 to 1.29)	1.31 (1.26 to 1.37)	205,288,899	13,768	0.93 (0.86 to 1.01)	1.04 (0.97 to 1.12)
	Dose 3	62,511,487	3875	1.19 (1.13 to 1.25)	1.32 (1.26 to 1.40)	62,511,487	3875	0.89 (0.82 to 0.97)	1.05 (0.96 to 1.14)
Menstruation absent/scanty	Unvaccinated	567,184,386	7111	1.00	1.00	456,450,497	5953	1.00	1.00
	Dose 1	38,588,311	485	1.17 (1.04 to 1.32)	1.19 (1.06 to 1.34)	38,588,311	485	0.97 (0.82 to 1.13)	1.03 (0.88 to 1.21)
	Dose 2	226,152,586	2622	1.05 (0.96 to 1.15)	1.23 (1.12 to 1.35)	226,152,586	2622	0.83 (0.71 to 0.98)	1.04 (0.89 to 1.22)
	Dose 3	69,414,402	639	0.91 (0.81 to 1.02)	1.15 (1.02 to 1.30)	69,414,402	639	0.72 (0.60 to 0.87)	0.98 (0.81 to 1.17)
Menstruation excessive	Unvaccinated	567,122,438	7791	1.00	1.00	456,347,119	6452	1.00	1.00
	Dose 1	38,574,154	625	1.25 (1.13 to 1.39)	1.23 (1.13 to 1.37)	38,574,154	625	1.03 (0.89 to 1.19)	1.04 (0.90 to 1.21)
	Dose 2	225,548,068	3,665	1.14 (1.06 to 1.23)	1.26 (1.16 to 1.36)	225,548,068	3,665	0.90 (0.77 to 1.04)	1.03 (0.89 to 1.20)
	Dose 3	68,995,389	1124	1.14 (1.04 to 1.26)	1.34 (1.22 to 1.48)	68,995,389	1124	0.89 (0.76 to 1.05)	1.09 (0.93 to 1.29)
Irregular/frequent menstrual periods	Unvaccinated	553,425,740	18,531	1.00	1.00	445,227,651	15,705	1.00	1.00
	Dose 1	37,157,996	1341	1.25 (1.16 to 1.34)	1.20 (1.12 to 1.29)	37,157,996	1341	0.97 (0.88 to 1.07)	0.98 (0.89 to 1.08)
	Dose 2	216,779,335	8658	1.28 (1.22 to 1.35)	1.32 (1.25 to 1.40)	216,779,335	8658	0.95 (0.86 to 1.05)	1.04 (0.94 to 1.15)
	Dose 3	66,119,072	2420	1.21 (1.13 to 1.30)	1.31 (1.23 to 1.40)	66,119,072	2420	0.90 (0.80 to 1.00)	1.02 (0.92 to 1.14)
Intermenstrual bleeding	Unvaccinated	571,951,296	3230	1.00	1.00	460,109,635	2719	1.00	1.00
	Dose 1	39,119,337	236	1.27 (1.08 to 1.51)	1.23 (1.04 to 1.46)	39,119,337	236	1.03 (0.82 to 1.31)	1.05 (0.83 to 1.32)
	Dose 2	228,982,205	1288	1.11 (0.97 to 1.26)	1.15 (1.01 to 1.31)	228,982,205	1288	0.87 (0.68 to 1.10)	0.95 (0.75 to 1.21)
	Dose 3	70,142,927	399	1.11 (0.95 to 1.30)	1.23 (1.04 to 1.44)	70,142,927	399	0.87 (0.67 to 1.13)	1.01 (0.78 to 1.32)

Adjusted for age at start of follow-up, income, education, marital status, region of birth, endometriosis, polycystic ovarian syndrome, diabetes mellitus, thyroid disorders and confirmed Covid-19

**Table 4:** Time since first dose of covid-19 vaccination and bleeding disturbances among women between 20 and 40 years of age

Outcome	Exposure group	Full sample				Excluding those who remain unvaccinated at the end of follow-up			
		Follow-up time in days	N Cases	Unadjusted HR (95% CI)	Adjusted HR (95% CI)	Follow-up time in days	N Cases	Unadjusted HR (95% CI)	Adjusted HR (95% CI)
Any menstrual bleeding disturbance	Unvaccinated	534,178,634	32,420	1.00	1.00	429,781,852	27,441	1.00	1.00
	0–60 days	27,517,370	1592	1.11 (1.04 to 1.19)	1.13 (1.06 to 1.21)	27,517,370	1592	0.95 (0.88 to 1.02)	0.99 (0.92 to 1.07)
	61–120 days	27,521,083	1996	1.23 (1.16 to 1.31)	1.27 (1.19 to 1.35)	27,521,083	1996	1.00 (0.91 to 1.09)	1.08 (0.99 to 1.17)
	121–180 days	27,735,306	2009	1.26 (1.18 to 1.35)	1.31 (1.23 to 1.40)	27,735,306	2009	0.97 (0.88 to 1.07)	1.08 (0.98 to 1.19)
	More than 180 days	220,224,478	14,296	1.29 (1.23 to 1.36)	1.38 (1.31 to 1.45)	220,224,478	14,296	0.97 (0.88 to 1.08)	1.13 (1.02 to 1.25)
Menstruation absent/scanty	Unvaccinated	567,184,386	7111	1.00	1.00	456,450,497		1.00	1.00
	0–60 days	29,888,074	359	1.09 (0.95 to 1.26)	1.17 (1.02 to 1.34)	29,888,074	359	0.93 (0.79 to 1.10)	1.03 (0.88 to 1.21)
	61–120 days	29,977,330	437	1.11 (0.96 to 1.27)	1.22 (1.06 to 1.40)	29,977,330	437	0.86 (0.72 to 1.04)	1.03 (0.85 to 1.24)
	121–180 days	30,302,938	413	1.09 (0.94 to 1.25)	1.24 (1.07 to 1.42)	30,302,938	413	0.84 (0.68 to 1.04)	1.07 (0.86 to 1.31)
	More than 180 days	243,986,957	2537	1.03 (0.93 to 1.14)	1.23 (1.10 to 1.36)	243,986,957	2537	0.79 (0.63 to 0.99)	1.11 (0.88 to 1.38)
Menstruation excessive	Unvaccinated	567,122,438	7791	1.00	1.00	456,347,119	6452	1.00	1.00
	0–60 days	29,840,998	423	1.10 (0.97 to 1.24)	1.14 (1.00 to 1.29)	29,840,998	423	0.98 (0.84 to 1.14)	1.03 (0.89 to 1.20)
	61–120 days	29,925,316	485	1.14 (1.00 to 1.29)	1.21 (1.06 to 1.37)	29,925,316	485	0.97 (0.82 to 1.15)	1.06 (0.89 to 1.26)
	121–180 days	30,253,485	481	1.11 (0.97 to 1.26)	1.20 (1.05 to 1.36)	30,253,485	481	0.89 (0.73 to 1.08)	1.00 (0.83 to 1.38)
	More than 180 days	243,097,812	4025	1.21 (1.11 to 1.32)	1.35 (1.23 to 1.48)	243,097,812	4025	0.95 (0.77 to 1.17)	1.13 (0.92 to 1.38)
Irregular/frequent menstrual periods	Unvaccinated	553,425,740	18,531	1.00	1.00	445,227,651	15,705	1.00	1.00
	0–60 days	28,857,212	921	1.09 (1.00 to 1.18)	1.09 (1.00 to 1.19)	28,857,212	921	0.91 (0.83 to 1.01)	0.95 (0.86 to 1.05)
	61–120 days	28,904,802	1235	1.31 (1.21 to 1.42)	1.32 (1.22 to 1.44)	28,904,802	1235	1.05 (0.94 to 1.18)	1.12 (1.00 to 1.25)
	121–180 days	29,176,873	1253	1.29 (1.19 to 1.40)	1.31 (1.21 to 1.43)	29,176,873	1253	0.99 (0.87 to 1.12)	1.07 (0.95 to 1.22)
	More than 180 days	233,117,516	9010	1.33 (1.25 to 1.41)	1.38 (1.30 to 1.47)	233,117,516	9010	1.00 (0.87 to 1.14)	1.13 (0.99 to 1.29)
Intermenstrual bleeding	Unvaccinated	571,951,296	3230	1.00	1.00	460,109,635	2719	1.00	1.00
	0–60 days	30,207,549	152	1.04 (0.84 to 1.28)	1.04 (0.85 to 1.29)	30,207,549	152	0.93 (0.72 to 1.18)	0.96 (0.75 to 1.23)
	61–120 days	30,312,990	173	1.08 (0.87 to 1.33)	1.10 (0.89 to 1.36)	30,312,990	173	0.96 (0.72 to 1.28)	1.02 (0.77 to 1.36)
	121–180 days	30,662,236	174	1.18 (0.96 to 1.46)	1.22 (0.98 to 1.51)	30,662,236	174	1.05 (0.76 to 1.44)	1.14 (0.83 to 1.57)
	More than 180 days	247,061,694	1424	1.20 (1.04 to 1.40)	1.26 (1.08 to 1.46)	247,061,694	1424	1.10 (0.78 to 1.55)	1.24 (0.88 to 1.75)

Adjusted for age at start of follow-up, income, education, marital status, region of birth, endometriosis, polycystic ovarian syndrome, diabetes mellitus, thyroid disorders and confirmed Covid-19

### Self-controlled case series analysis of Covid-19 vaccination and risk of menstrual disturbances

In the self-controlled case series analysis (Table 5), we observed a modest increased risk of absent/scanty menstruation (IRR 1.19; 95% CI: 1.08, 1.31) and irregular/frequent menstrual periods (IRR 1.25; 95% CI: 1.18, 1.32) starting from 61 days after having been given dose 1. No increased risk was observed for the other menstrual disturbances. The self-controlled case-series analysis of dose 2 showed estimates of a slightly greater magnitude, and some evidence of an increased risk of absent/scanty menstrual bleeding and irregular/frequent menstrual bleeding starting from 31 days after being given dose 2 (Supplementary Table S4).

**Table 5:** Self-controlled case series of the first dose of covid-19 vaccination and bleeding disturbances among women between 20 and 40 years of age

Outcome	Time window around the first vaccine dose	Number at risk during the time window	Number of events during the time window	IRR (95% CI)
Any menstrual bleeding disturbance	180 days until 61 days before vaccination	18,516	3934	1.00
	60 − 31 days before vaccination	10,318	907	1.08 (1.01 to 1.16)
	0–30 days before vaccination	10,410	815	1.01 (0.93 to 1.09)
	0–30 days after vaccination	10,419	806	0.93 (0.86 to 1.01)
	31–60 days after vaccination	10,380	845	1.01 (0.94 to 1.09)
	61–180 days after vaccination	7307	3918	1.17 (1.12 to 1.22)
Menstruation absent/scanty	180 days before until 61 days before vaccination	3776	800	1.00
	0–60 days before vaccination	1958	330	0.99 (0.87 to 1.12)
	0–60 days after vaccination	1938	350	1.01 (0.89 to 1.15)
	61–180 days after vaccination	1480	808	1.19 (1.08 to 1.31)
Menstruation excessive	180 days until 61 days before vaccination	4774	1050	1.00
	60 − 31 days before vaccination	2669	243	1.09 (0.95 to 1.25)
	0–30 days before vaccination	2700	212	0.98 (0.85 to 1.14)
	0–30 days after vaccination	2683	229	0.99 (0.86 to 1.14)
	31–60 days after vaccination	2697	215	0.96 (0.83 to 1.11)
	61–180 days after vaccination	1949	963	1.08 (0.99 to 1.18)
Irregular/frequent menstrual periods	180 days until 61 days before vaccination	11,059	2265	1.00
	60 − 31 days before vaccination	6145	517	1.07 (0.98 to 1.18)
	0–30 days before vaccination	6153	509	1.09 (0.99 to 1.20)
	0–30 days after vaccination	6187	475	0.95 (0.86 to 1.05)
	31–60 days after vaccination	6168	494	1.03 (0.93 to 1.13)
	61–180 days after vaccination	4260	2402	1.25 (1.18 to 1.32)
Intermenstrual bleeding	180 days until 61 days before vaccination	1798	426	1.00
	60 − 31 days before vaccination	1018	94	1.04 (0.84 to 1.30)
	0–30 days before vaccination	1034	78	0.89 (0.70 to 1.13)
	0–30 days after vaccination	1026	86	0.92 (0.73 to 1.16)
	31–60 days after vaccination	1045	67	0.74 (0.57 to 0.96)
	61–180 days after vaccination	751	361	1.00 (0.87 to 1.15)

### Confirmed Covid-19

A total of 34% (*n* = 223,746) of women were registered with a positive test for SARS-CoV-2 by the end of follow-up. Overall, Covid-19 infection was associated with a modest increased risk of all menstrual bleeding disturbances (Supplementary Table S5). When we examined the risk according to time since infection, we found an increased risk of absent/scanty menstruation 180 days after infection, while an increased risk of excessive and irregular/frequent menstruation was seen immediately after infection, and an increased risk of intermenstrual bleeding was seen from 61 days after infection onwards (Supplementary Table S6). Our sensitivity analysis censoring the follow-up time at January 31st, 2022, attenuated the results (Supplementary Tables S7 and S8).

## Discussion

We observed a modestly increased risk of menstrual bleeding disturbances after Covid-19 vaccination, which completely attenuated after excluding the subgroup (14%) of women who remained unvaccinated throughout the pandemic (end of follow-up), highlighting a potential role of unmeasured confounding. Our self-controlled case series analysis indicated some evidence of a modestly increased risk of absent/scanty menstrual bleeding and irregular/frequent menstrual bleeding starting from 31 days after being given dose 2. We also observed an association between confirmed Covid-19 infection and menstrual disturbances.

The majority of studies investigating the association between Covid-19 vaccination and menstrual health evaluated the cycle length as the outcome, as it is a well-defined and easy to track outcome [3]. Among the studies with a prospective data collection on this topic, two U.S. Studies including 4,000 and 20,000 individuals, reported a transient increase in cycle length following vaccination [21, 24]. We were unfortunately not able to replicate these associations as we did not have information on cycle length. Substantially fewer studies have attempted to investigate other altered menstrual patterns [3, 4]. Three prospective studies from the U.S. and U.K. provided no evidence of altered menstrual cycle regularity after Covid-19 vaccination [25–27]. Additionally, the registry-based study from Sweden also provided no evidence that Covid-19 vaccination was associated with new registrations of menstrual cycle irregularity [6]. This seems to be in line with what we observed in our study. With regard to menstrual flow, the evidence is very mixed. The largest prospective study currently available (with 7501 vaccinated and 2154 unvaccinated individuals), indicated no difference in the number of days with heavy menstrual bleedings [28]. Other studies have reported no changes in self-reported menstrual flow following vaccination as we observed in the current study [29–31], and an increased risk of both “heavier” and “lighter” menstrual flow [22, 32–34], further complicating the interpretation of the evidence.

Our findings from the survival analysis were fully attenuated after excluding those who remained unvaccinated. Women who chose never to vaccinate through the pandemic are likely to be different in several unmeasured ways compared to the majority of women who at some point received covid-19 vaccination. Thus, we believe there is less unmeasured confounding when we compare risk after vaccination to risk before vaccination in a comparison group of women who later became vaccinated. The full attenuation of associations when excluding the smaller group of never-vaccinated women supports the possibility of residual confounding as suggested by some previous studies. We did observe a modest increased risk of some menstrual bleeding disturbances after Covid-19 vaccination in the self-controlled case series analysis, however, these results may reflect that women could have been more likely to seek health care after vaccination as this topic was covered in the media. An increased tendency to seek health care such a this would increase the likelihood of being registered with menstrual bleeding disturbances after vaccination compared to before vaccination.

mRNA technology (including its use in vaccines against Covid-19) has proved hugely successful due to rapid advances in biotechnology and molecular medicine, resulting in optimism regarding its potential usefulness to combat a range of diseases [35]. However, we still have a somewhat limited understanding of any potential side-effects of mRNA vaccines. Due to mass vaccination campaigns during the Covid-19 pandemic, and the resulting high proportion of individuals who have now received these vaccines, substantial efforts have been made to research potential side-effects. These efforts have reassured the global community that there are unlikely to be any serious side-effects of mRNA vaccines against Covid-19. Despite this, a better understanding of potential milder side-effects, due to the complex impact of the immune-activation on different organ systems, seems warranted. Our reassuring findings that mRNA Covid-19 vaccines are unlikely to impact the risk of menstrual bleeding disturbances are therefore important.

Substantially fewer studies have examined the risk of menstrual bleeding disturbances according to infection with Covid-19 [30, 36–41]. These studies have indicated some evidence of an increased risk of menstrual cycle irregularity [36], heavier menstrual bleedings [30], inter-menstrual bleedings [30], and potential decline in ovarian reserve [39]. Two studies that were restricted to individuals who had experienced Covid-19, reported more menstrual bleeding disturbances among women who had more severe COVID-19 symptoms [38, 40]. Overall, our findings are in line with previous studies indicating a modestly increased risk of menstrual bleeding disturbances after Covid-19 infection.

Important strengths of our study include the nationwide population-based design, the prospective registrations of menstrual disturbances by general practitioners, and our ability to evaluate several different menstrual disturbances. The estimated rate of menstrual disturbances in the Swedish study was 1407 per 100,000 person years [6]. In comparison, the rate was 2282 per 100,000 person years in our study, highlighting that we captured substantially more registrations than this previous study. Another important strength of our study is the inclusion of two different analytical approaches for the investigation of the association with vaccination. While the Cox-analysis approach evaluated new-onset of menstrual disturbances, by excluding women with a pre-existing history of menstrual disturbances two years prior to the start of follow-up in the model, the self-controlled case-series compared the women’s likelihood of being registered with a menstrual bleeding disturbance after compared to before vaccination. While a self-controlled case-series analysis might have been substantially less biased while the pandemic was ongoing, it has some assumptions as described in the methods which might not necessarily be strictly met. Specifically, one registration of a menstrual bleeding disturbance is indeed likely to affect the probability of having a second registration. In contrast, as we conducted our analyses towards the end of the pandemic, and were therefore able to conduct the survival analysis excluding those who remained unvaccinated at the end of follow-up, we believe this analysis might actually be the least biased.

There are also some limitations worth noting. We were only able to capture menstrual bleeding disturbances resulting in contact with a general practitioner. Notably, the overwhelming majority of Norwegian citizens are registered with a primary care physician, and women have to be referred by their primary care physician if they want to see a gynecologist. A minority of women might have by-passed this system and gone directly to a privately practicing gynecologist outside the public health-care system. This could have resulted in an underestimation of the associations of interest. The timing of this consultation may also differ from the onset of menstrual disturbances and vary according to both infection and vaccination. The national registries do not contain information on lifestyle characteristics, diet or measures of everyday stress, which may impact menstrual bleeding disturbances. This could have led us to overestimate the associations. Some of the pre-existing conditions adjusted for, such as PCOS and endometriosis, are likely to be under-recorded in the patient registry resulting in misclassification. We were also not able to explore adjustment for women’s patterns of all-cause health-care use (such as for example the frequency of consultations during the year before start of follow-up), as we only had information on contacts with health-care services for a subgroup of conditions to answer our research question. We were further unable to identify women who were not menstruating, for example due to use of contraceptives, premature ovarian failure, oophorectomy, or hysterectomy. A small proportion of the women included in the study are also likely to be to be perimenopausal, making them more likely to experience irregular and altered menstrual bleedings. This could potentially lead us to underestimate results if these women are also more likely to be exposed to infection with or vaccination against Covid-19. Our findings are also only generalizable to populations with similar demographic and social characteristics. Particularly, our findings also may also not be generalizable to countries where Covid-19 vaccines other than the mRNA vaccines were an important part of the vaccination program, and countries without universal health-care systems.

## Conclusion

We observed a modestly increased risk of menstrual bleeding disturbances after Covid-19 vaccination. This appeared to reflect a role of unmeasured confounding, as indicated by the attenuation of the associations in the analyses excluding the subgroup of women who remained unvaccinated throughout the pandemic and at the end of follow-up from the reference group.

## Electronic Supplementary Material

Below is the link to the electronic supplementary material.

Supplementary Material 1
