# Supplementary material for: Covid-19 vaccination and menstrual bleeding disturbances among women of fertile age: a Norwegian registry study
Source: Eur J Epidemiol. 2024 Nov 6;39(10):1127–38. doi: 10.1007/s10654-024-01170-0 (PMC11599392; doi:10.1007/s10654-024-01170-0)
Supplement: Supplementary file 1 — Supplementary Material 1 [file 10654_2024_1170_MOESM1_ESM.docx]

**Supplemental Online Content**

**Online Methods**

**Supplementary Table S1.** Types of covid-19 vaccination and bleeding disturbances among women of fertile age (20-40 years of age)

**Supplementary Table S2.** Covid-19 vaccination and bleeding disturbances among women of fertile age (20-40 years of age) stratified by time period

**Supplementary Table S3.** Covid-19 vaccination and bleeding disturbances among women between 20 and 40 years of ages sensitivity analysis stratified by whether or not they had experienced an infection with Covid-19 by the end of the follow-up period

**Supplementary Table S4.** Self-controlled case series of the second dose of covid-19 vaccination and bleeding disturbances among women between 20 and 40 years of age

**Supplementary Table S5.** Confirmed Covid-19 and bleeding disturbances among women of fertile age (20-40 years of age)

**Supplementary Table S6.** Time since confirmed Covid-19 and bleeding disturbances among women of fertile age (20-40 years of age)

**Supplementary Table S7.** Confirmed Covid-19 and bleeding disturbances among women of fertile age (20-40 years of age) sensitivity analysis restricting the follow-up to January 31^st^ 2022

**Supplementary Table S8.** Time since confirmed Covid-19 and bleeding disturbances among women of fertile age (20-40 years of age) sensitivity analysis restricting the follow-up to January 31^st^ 2022

**Online Methods**

**Data sources and linkages in Norway**

*The Emergency Preparedness Register for Covid-19*

Data in this study were provided through the Emergency preparedness register for Covid-19 (Beredt C19) administered by the Norwegian Institute of Public Health, according to the Health Preparedness Act §2 to 4. This registry was established in 2020 to provide authorities with up to date information on prevalence, causal relationships, and consequences of the Covid19 epidemic in Norway. Beredt C19 includes information already collected in the healthcare service, national health registries and administrative registers with information about the Norwegian population. The data subjects' right is safeguarded as they can contact the data controller for all different sources included in Beredt C19 in the usual way. Through Beredt C19 we used data from the following sources:

*The Norwegian Immunisation Register (SYSVAK)*

SYSVAK is a register of vaccines in the Norwegian vaccination program, with mandatory registrations of all Covid-19 vaccinations (dates and type).

*Norwegian Surveillance System for Communicable Diseases (MSIS)*

There is mandatory reporting of selected infectious diseases to this National Health register. Reporting of all positive PCR-tests for Covid-19 tests is mandatory, and this register contains date of testing and test results.

*Statistics Norway (SSB)*

Administrative data is mandatorily reported to Statistics Norway. We used information from this database on household income in 2018, type of education and years of education completed by 2019.

*The Medical Birth Registry of Norway (MBRN)*

The Norwegian national birth registry includes information on all pregnancies ending in gestational week 12 or later. The registry includes information on birth outcomes in addition to maternal background characteristics, health during pregnancy, pregnancy outcomes and neonatal health.

*The Norwegian Patient Registry (NPR)*

The Norwegian Patient Registry includes individual level information on all contacts with specialist health-care services. Information registered includes admission and discharge dates, and diagnostic codes during the hospital stay. These discharge codes are coded according to the International Classification of Diseases version 10.

*Norwegian Registry of Primary Health Care (KPR)*

This registry includes the date, the reimbursement code for payment of services, and medical diagnoses for all consultations in the primary care services. This includes all contact with general practitioners. The diagnoses are coded according to the International Classification of Primary Care version 2.

**Supplementary Table S1. Types of covid-19 vaccination and bleeding disturbances among women between 20 and 40 years of age**

| **Outcome** | **Exposure group** | **Full sample** | | | | **Excluding those who remain unvaccinated at the end of follow-up** | | | |
| --- | --- | --- | --- | --- | --- | --- | --- | --- | --- |
|  |  | **Follow-up time in days** | **N Cases** | **Unadjusted**  **HR (95% CI)** | **Adjusted**  **HR (95% CI)** | **Follow-up time in days** | **N Cases** | **Unadjusted**  **HR (95% CI)** | **Adjusted**  **HR (95% CI)** |
| Any menstrual bleeding disturbance | Unvaccinated | 534178634 | 32420 | 1.00 | 1.00 | 429781852 | 27441 | 1.00 | 1.00 |
|  | mRNA-1273 (Moderna) | 47879421 | 3131 | 1.23 (1.17 to 1.30) | 1.29 (1.23 to 1.36) | 47879421 | 3131 | 0.96 (0.89 to 1.04) | 1.02 (0.95 to 1.11) |
|  | BNT162b2 (Pfizer-BioNTech) | 175210480 | 11488 | 1.24 (1.19 to 1.29) | 1.30 (1.25 to 1.35) | 175210480 | 11488 | 0.96 (0.90 to 1.03) | 1.03 (0.96 to 1.11) |
|  | Mixed | 79917336 | 5274 | 1.25 (1.19 to 1.30) | 1.27 (1.22 to 1.33) | 79917336 | 5274 | 0.97 (0.89 to 1.04) | 1.00 (0.93 to 1.08) |
| Menstruation absent/scanty | Unvaccinated | 567184386 | 7111 | 1.00 | 1.00 | 456450497 | 5953 | 1.00 | 1.00 |
|  | mRNA-1273 (Moderna) | 52462516 | 609 | 1.10 (0.98 to 1.23) | 1.23 (1.10 to 1.37) | 52462516 | 609 | 0.94 (0.79 to 1.11) | 1.04 (0.88 to 1.23) |
|  | BNT162b2 (Pfizer-BioNTech) | 193695097 | 2130 | 1.04 (0.95 to 1.13) | 1.21 (1.11 to 1.33) | 193695097 | 2130 | 0.89 (0.76 to 1.03) | 1.04 (0.89 to 1.21) |
|  | Mixed | 87997686 | 1007 | 1.09 (0.98 to 1.20) | 1.19 (1.08 to 1.32) | 87997686 | 1007 | 0.92 (0.78 to 1.09) | 1.01 (0.86 to 1.19) |
| Menstruation excessive | Unvaccinated | 567122438 | 7791 | 1.00 | 1.00 | 456347119 | 6452 | 1.00 | 1.00 |
|  | mRNA-1273 (Moderna) | 52497350 | 827 | 1.13 (1.02 to 1.24) | 1.25 (1.14 to 1.38) | 52497350 | 827 | 0.93 (0.80 to 1.08) | 1.02 (0.88 to 1.19) |
|  | BNT162b2 (Pfizer-BioNTech) | 192766457 | 3178 | 1.18 (1.09 to 1.28) | 1.28 (1.19 to 1.38) | 192766457 | 3178 | 0.98 (0.85 to 1.12) | 1.05 (0.92 to 1.21) |
|  | Mixed | 87853804 | 1409 | 1.15 (1.05 to 1.25) | 1.22 (1.12 to 1.33) | 87853804 | 1409 | 0.94 (0.81 to 1.09) | 0.99 (0.86 to 1.15) |
| Irregular/frequent menstrual periods | Unvaccinated | 553425740 | 18531 | 1.00 | 1.00 | 445227651 | 15705 | 1.00 | 1.00 |
|  | mRNA-1273 (Moderna) | 50493870 | 1898 | 1.23 (1.15 to 1.31) | 1.26 (1.18 to 1.35) | 50493870 | 1898 | 0.93 (0.84 to 1.03) | 0.98 (0.88 to 1.08) |
|  | BNT162b2 (Pfizer-BioNTech) | 185140585 | 7231 | 1.27 (1.21 to 1.34) | 1.31 (1.24 to 1.38) | 185140585 | 7231 | 0.97 (0.88 to 1.06) | 1.02 (0.93 to 1.12) |
|  | Mixed | 84421948 | 3290 | 1.27 (1.20 to 1.35) | 1.27 (1.20 to 1.35) | 84421948 | 3290 | 0.96 (0.87 to 1.06) | 0.99 (0.89 to 1.09) |
| Intermenstrual bleeding | Unvaccinated | 571951296 | 3230 | 1.00 | 1.00 | 460109635 | 2719 | 1.00 | 1.00 |
|  | mRNA-1273 (Moderna) | 53220535 | 307 | 1.16 (0.99 to 1.36) | 1.20 (1.02 to 1.41) | 53220535 | 307 | 0.97 (0.76 to 1.24) | 1.02 (0.79 to 1.30) |
|  | BNT162b2 (Pfizer-BioNTech) | 195884927 | 1100 | 1.13 (0.99 to 1.28) | 1.17 (1.03 to 1.34) | 195884927 | 1100 | 0.95 (0.75 to 1.19) | 1.00 (0.80 to 1.25) |
|  | Mixed | 89139007 | 516 | 1.17 (1.01 to 1.35) | 1.17 (1.01 to 1.36) | 89139007 | 516 | 0.97 (0.76 to 1.24) | 0.99 (0.78 to 1.27) |

Adjusted for age at start of follow-up, income, education, marital status, region of birth, endometriosis, polycystic ovarian syndrome, diabetes mellitus, thyroid disorders and confirmed Covid-19.

**Supplementary Table S2. Covid-19 vaccination and bleeding disturbances among women between 20 and 40 years of age stratified by time period**

| **Outcome** | **Time period** | **Exposure group** | **Full sample** | | | | **Excluding those who remain unvaccinated at the end of follow-up** | | | |
| --- | --- | --- | --- | --- | --- | --- | --- | --- | --- | --- |
|  |  |  | **Follow-up time in days** | **N Cases** | **Unadjusted**  **HR (95% CI)** | **Adjusted**  **HR (95% CI)** | **Follow-up time in days** | **N Cases** | **Unadjusted**  **HR (95% CI)** | **Adjusted**  **HR (95% CI)** |
| Any menstrual bleeding disturbance | Before July 1st 2021 | Unvaccinated | 487170380 | 30089 | 1.00 | 1.00 | 421208120 | 26955 | 1.00 | 1.00 |
|  |  | Vaccinated | 7803750 | 520 | 1.05 (0.96 to 1.16) | 1.10 (1.00 to 1.21) | 7803750 | 520 | 1.00 (0.91 to 1.10) | 1.06 (0.96 to 1.16) |
|  | After July 1st 2021 | Unvaccinated | 47008254 | 2331 | 1.00 | 1.00 | 8573732 | 486 | 1.00 | 1.00 |
|  |  | Vaccinated | 295194487 | 19373 | 1.28 (1.23 to 1.34) | 1.34 (1.28 to 1.40) | 295194487 | 19373 | 0.92 (0.83 to 1.02) | 0.98 (0.88 to 1.08) |
| Menstruation absent/scanty | Before July 1st 2021 | Unvaccinated | 516227370 | 6580 | 1.00 | 1.00 | 447115822 | 5838 | 1.00 | 1.00 |
|  |  | Vaccinated | 8481966 | 104 | 0.99 (0.81 to 1.22) | 1.10 (0.89 to 1.35) | 8481966 | 104 | 0.94 (0.77 to 1.16) | 1.05 (0.85 to 1.29) |
|  | After July 1st 2021 | Unvaccinated | 50957016 | 531 | 1.00 | 1.00 | 9334675 | 115 | 1.00 | 1.00 |
|  |  | Vaccinated | 325673333 | 3642 | 1.07 (0.98 to 1.18) | 1.25 (1.14 to 1.38) | 325673333 | 3642 | 0.95 (0.69 to 1.06) | 1.00 (0.81 to 1.24) |
| Menstruation excessive | Before July 1st 2021 | Unvaccinated | 516211982 | 7123 | 1.00 | 1.00 | 447009860 | 6324 | 1.00 | 1.00 |
|  |  | Vaccinated | 8445944 | 140 | 1.06 (0.89 to 1.27) | 1.13 (0.94 to 1.35) | 8445944 | 140 | 1.03 (0.85 to 1.23) | 1.08 (0.90 to 1.30) |
|  | After July 1st 2021 | Unvaccinated | 50910456 | 668 | 1.00 | 1.00 | 9337259 | 128 | 1.00 | 1.00 |
|  |  | Vaccinated | 324671667 | 5274 | 1.19 (1.09 to 1.29) | 1.32 (1.21 to 1.43) | 324671667 | 5274 | 0.89 (0.73 to 1.10) | 0.99 (0.81 to 1.21) |
| Irregular/frequent menstrual periods | Before July 1st 2021 | Unvaccinated | 504116201 | 17111 | 1.00 | 1.00 | 436217486 | 15394 | 1.00 | 1.00 |
|  |  | Vaccinated | 8171805 | 316 | 1.11 (0.99 to 1.26) | 1.15 (1.02 to 1.30) | 8171805 | 316 | 1.06 (0.94 to 1.20) | 1.11 (0.98 to 1.25) |
|  | After July 1st 2021 | Unvaccinated | 49309539 | 1420 | 1.00 | 1.00 | 9010165 | 311 | 1.00 | 1.00 |
|  |  | Vaccinated | 311884598 | 12103 | 1.30 (1.23 to 1.38) | 1.31 (1.24 to 1.39) | 311884598 | 12103 | 0.85 (0.75 to 0.97) | 0.89 (0.78 to 1.01) |
| Intermenstrual bleeding | Before July 1st 2021 | Unvaccinated | 520342150 | 2990 | 1.00 | 1.00 | 450655841 | 2674 | 1.00 | 1.00 |
|  |  | Vaccinated | 8551151 | 50 | 0.98 (0.73 to 1.33) | 1.02 (0.75 to 1.38) | 8551151 | 50 | 0.94 (0.69 to 1.27) | 0.98 (0.72 to 1.33) |
|  | After July 1st 2021 | Unvaccinated | 51609146 | 240 | 1.00 | 1.00 | 9453794 | 45 | 1.00 | 1.00 |
|  |  | Vaccinated | 329693318 | 1873 | 1.18 (1.03 to 1.35) | 1.22 (1.05 to 1.40) | 329693318 | 1873 | 0.97 (0.69 to 1.37) | 1.03 (0.73 to 1.45) |

Adjusted for age at start of follow-up, income, education, marital status, region of birth, endometriosis, polycystic ovarian syndrome, diabetes mellitus, thyroid disorders and confirmed Covid-19.

**Supplementary Table S3. Covid-19 vaccination and bleeding disturbances among women between 20 and 40 years of ages sensitivity analysis stratified by whether or not they had experienced an infection with Covid-19 by the end of the follow-up period**

| **Population** | **Outcome** | **No infection by the end of follow-up** | | | | | **At least one infection by the end of follow-up** | | | |
| --- | --- | --- | --- | --- | --- | --- | --- | --- | --- | --- |
|  |  | **Exposure group** | **Follow-up time in days** | **N Cases** | **Unadjusted**  **HR (95% CI)** | **Adjusted**  **HR (95% CI)** | **Follow-up time in days** | **N Cases** | **Unadjusted**  **HR (95% CI)** | **Adjusted**  **HR (95% CI)** |
| Full sample | Any menstrual bleeding disturbance | Unvaccinated | 352105652 | 19743 | 1.00 | 1.00 | 182072982 | 12677 | 1.00 | 1.00 |
|  |  | Vaccinated | 204012589 | 12560 | 1.46 (1.38 to 1.54) | 1.47 (1.39 to 1.55) | 98985648 | 7333 | 1.07 (1.01 to 1.13) | 1.12 (1.06 to 1.19) |
|  | Menstruation absent/scanty | Unvaccinated | 372294187 | 4413 | 1.00 | 1.00 | 194890199 | 2698 | 1.00 | 1.00 |
|  |  | Vaccinated | 224045628 | 2410 | 1.32 (1.17 to 1.48) | 1.48 (1.32 to 1.67) | 110109671 | 1336 | 0.85 (0.76 to 0.96) | 0.96 (0.85 to 1.08) |
|  | Menstruation excessive | Unvaccinated | 372360814 | 4628 | 1.00 | 1.00 | 194761624 | 3163 | 1.00 | 1.00 |
|  |  | Vaccinated | 223482889 | 3335 | 1.35 (1.22 to 1.50) | 1.40 (1.26 to 1.56) | 109634722 | 2079 | 1.04 (0.94 to 1.16) | 1.14 (1.02 to 1.26) |
|  | Irregular/frequent menstrual periods | Unvaccinated | 363991136 | 11242 | 1.00 | 1.00 | 189434604 | 7289 | 1.00 | 1.00 |
|  |  | Vaccinated | 215037767 | 7810 | 1.48 (1.38 to 1.58) | 1.46 (1.36 to 1.57) | 105018636 | 4609 | 1.10 (1.02 to 1.18) | 1.14 (1.06 to 1.22) |
|  | Intermenstrual bleeding | Unvaccinated | 375316683 | 1943 | 1.00 | 1.00 | 196634613 | 1287 | 1.00 | 1.00 |
|  |  | Vaccinated | 226751523 | 1184 | 1.29 (1.09 to 1.53) | 1.30 (1.09 to 1.54) | 111492946 | 739 | 1.05 (0.88 to 1.25) | 1.07 (0.89 to 1.28) |
| Excluding those who remained unvaccinated at the end of follow-up | Any menstrual bleeding disturbance | Unvaccinated | 287324947 | 17530 | 1.00 | 1.00 | 142456905 | 9911 | 1.00 | 1.00 |
|  |  | Vaccinated | 204012589 | 12560 | 0.98 (0.90 to 1.07) | 1.03 (0.95 to 1.13) | 98985648 | 7333 | 0.97 (0.6 to 1.08) | 1.02 (0.91 to 1.14) |
|  | Menstruation absent/scanty | Unvaccinated | 425078402 | 5058 | 1.00 | 1.00 | 213150122 | 2766 | 1.00 | 1.00 |
|  |  | Vaccinated | 103449907 | 1235 | 0.97 /0.85 to 1.08) | 1.01 (0.90 to 1.14) | 46927365 | 640 | 0.86 (0.73 to 1.00) | 0.90 (0.77 to 1.05) |
|  | Menstruation excessive | Unvaccinated | 412713582 | 14048 | 1.00 | 1.00 | 207962972 | 8204 | 1.00 | 1.00 |
|  |  | Vaccinated | 99620999 | 3759 | 1.02 (0.95 to 1.09) | 1.03 (0.96 to 1.10) | 44986501 | 2113 | 0.89 (0.82 to 0.97) | 0.90 (0.83 to 0.98) |
|  | Irregular/frequent menstrual periods | Unvaccinated | 424701238 | 5873 | 1.00 | 1.00 | 214754893 | 3581 | 1.00 | 1.00 |
|  |  | Vaccinated | 103221870 | 1507 | 1.01 (0.91 to 1.11) | 1.02 (0.92 to 1.13) | 46786729 | 905 | 0.93 (0.82 to 1.06) | 0.95 (0.84 to 1.08) |
|  | Intermenstrual bleeding | Unvaccinated | 429286322 | 2336 | 1.00 | 1.00 | 217459798 | 1423 | 1.00 | 1.00 |
|  |  | Vaccinated | 104712360 | 566 | 1.13 (0.96 to 1.34) | 1.15 (0.96 to 1.36) | 47516832 | 317 | 0.97 (0.79 to 1.21) | 0.95 (0.76 to 1.18) |

Adjusted for age at start of follow-up, income, education, marital status, region of birth, endometriosis, polycystic ovarian syndrome, diabetes mellitus, and thyroid disorders.

**Supplementary Table S4. Self-controlled case series of the second dose of covid-19 vaccination and bleeding disturbances among women between 20 and 40 years of age**

| **Outcome** | **Time window around the second vaccine dose** | **Number at risk during the time window** | **Number of events during the time window** | **IRR (95% CI)** |
| --- | --- | --- | --- | --- |
| Any menstrual bleeding disturbance | 180 days until 61 days before vaccination | 13856 | 2718 | 1.00 |
|  | 60-31 days before vaccination | 7735 | 552 | 0.95 (0.87 to 1.05) |
|  | 0-30 days before vaccination | 7724 | 563 | 1.01 (0.92 to 1.10) |
|  | 0-30 days after vaccination | 7638 | 649 | 1.09 (1.00 to 1.18) |
|  | 31-60 days after vaccination | 7602 | 685 | 1.18 (1.09 to 1.29) |
|  | 61-180 days after vaccination | 5167 | 3120 | 1.35 (1.28 to 1.42) |
| Menstruation absent/scanty | 180 days before until 61 days before vaccination | 2689 | 529 | 1.00 |
|  | 0-60 days before vaccination | 1412 | 197 | 0.89 (0.76 to 1.05) |
|  | 0-60 days after vaccination | 1322 | 287 | 1.25 (1.09 to 1.45) |
|  | 61-180 days after vaccination | 1013 | 596 | 1.32 (1.18 to 1.49) |
| Menstruation excessive | 180 days until 61 days before vaccination | 3527 | 723 | 1.00 |
|  | 60-31 days before vaccination | 1973 | 152 | 0.99 (0.83 to 1.18) |
|  | 0-30 days before vaccination | 1967 | 158 | 1.06 (0.89 to 1.26) |
|  | 0-30 days after vaccination | 1960 | 165 | 1.04 (0.88 to 1.23) |
|  | 31-60 days after vaccination | 1955 | 170 | 1.11 (0.94 to 1.31) |
|  | 61-180 days after vaccination | 1368 | 757 | 1.23 (1.11 to 1.36) |
| Irregular/frequent menstrual periods | 180 days until 61 days before vaccination | 8534 | 1568 | 1.00 |
|  | 60-31 days before vaccination | 4702 | 349 | 1.05 (0.93 to 1.17) |
|  | 0-30 days before vaccination | 4718 | 333 | 1.03 (0.92 to 1.16) |
|  | 0-30 days after vaccination | 4659 | 392 | 1.14 (1.02 to 1.27) |
|  | 31-60 days after vaccination | 4640 | 411 | 1.23 (1.11 to 1.37) |
|  | 61-180 days after vaccination | 3053 | 1998 | 1.50 (1.40 to 1.60) |
| Intermenstrual bleeding | 180 days until 61 days before vaccination | 322 | 16 | 1.00 |
|  | 60-31 days before vaccination | 166 | 3 | 0.88 (0.26 to 3.02) |
|  | 0-30 days before vaccination | 164 | 5 | 1.52 (0.56 to 4.15) |
|  | 0-30 days after vaccination | 163 | 6 | 1.71 (0.67 to 4.36) |
|  | 31-60 days after vaccination | 166 | 3 | 0.88 (0.26 to 3.02) |
|  | 61-180 days after vaccination | 154 | 15 | 1.10 (0.54 to 2.23) |

**Supplementary Table S5. Confirmed Covid-19 and bleeding disturbances among women between 20 and 40 years of age**

| **Outcome** | **Exposure group** | **Follow-up time in days** | **N Cases** | **Unadjusted**  **HR (95% CI)** | **Adjusted**  **HR (95% CI)** |
| --- | --- | --- | --- | --- | --- |
| Any menstrual bleeding disturbance | No infection | 752316556 | 46440 | 1.00 | 1.00 |
|  | Infected | 84860315 | 5873 | 1.17 (1.14 to 1.21) | 1.16 (1.13 to 1.20) |
| Menstruation absent/scanty | No infection | 806567718 | 9776 | 1.00 | 1.00 |
|  | Infected | 94771967 | 1081 | 1.15 (1.07 to 1.24) | 1.08 (1.01 to 1.17) |
| Menstruation excessive | No infection | 805863231 | 11459 | 1.00 | 1.00 |
|  | Infected | 94376818 | 1746 | 1.25 (1.18 to 1.33) | 1.21 (1.14 to 1.29) |
| Irregular/frequent menstrual periods | No infection | 783223773 | 27271 | 1.00 | 1.00 |
|  | Infected | 90258370 | 3679 | 1.17 (1.12 to 1.21) | 1.17 (1.12 to 1.22) |
| Intermenstrual bleeding | No infection | 814136510 | 4533 | 1.00 | 1.00 |
|  | Infected | 96059615 | 620 | 1.24 (1.12 to 1.37) | 1.24 (1.13 to 1.38) |

Adjusted for age at start of follow-up, income, education, marital status, region of birth, endometriosis, polycystic ovarian syndrome, diabetes mellitus, thyroid disorders and number of doses of vaccines against Covid-19.

**Supplementary Table S6. Time since confirmed Covid-19 and bleeding disturbances among women between 20 and 40 years of age**

| **Outcome** | **Exposure group** | **Follow-up time in days** | **N Cases** | **Unadjusted HR (95% CI)** | **Adjusted HR (95% CI)** |
| --- | --- | --- | --- | --- | --- |
| Any menstrual bleeding disturbance | No infection | 752316556 | 46440 | 1.00 | 1.00 |
|  | 0-60 days | 10431794 | 779 | 1.14 (1.06 to 1.23) | 1.13 (1.05 to 1.22) |
|  | 61-120 days | 10408956 | 715 | 1.19 (1.10 to 1.29) | 1.18 (1.09 to 1.28) |
|  | 121-180 days | 10328687 | 605 | 1.21 (1.11 to 1.32) | 1.20 (1.10 to 1.30) |
|  | More than 180 days | 53690878 | 3774 | 1.18 (1.13 to 1.22) | 1.16 (1.12 to 1.21) |
| Menstruation absent/scanty | No infection | 806567718 | 9776 | 1.00 | 1.00 |
|  | 0-60 days | 11530281 | 150 | 1.15 (0.96 to 1.36) | 1.10 (0.93 to 1.31) |
|  | 61-120 days | 11538707 | 120 | 1.00 (0.82 to 1.21) | 0.96 (0.79 to 1.17) |
|  | 121-180 days | 11484077 | 104 | 1.06 (0.86 to 1.30) | 1.01 (0.82 to 1.24) |
|  | More than 180 days | 60218902 | 707 | 1.20 (1.09 to 1.31) | 1.12 (1.02 to 1.23) |
| Menstruation excessive | No infection | 805863231 | 11459 | 1.00 | 1.00 |
|  | 0-60 days | 11493759 | 217 | 1.23 (1.07 to 1.42) | 1.20 (1.04 to 1.39) |
|  | 61-120 days | 11497689 | 200 | 1.22 (1.05 to 1.42) | 1.20 (1.03 to 1.39) |
|  | 121-180 days | 11437205 | 195 | 1.38 (1.19 to 1.61) | 1.34 (1.15 to 1.57) |
|  | More than 180 days | 59948165 | 1134 | 1.24 (1.15 to 1.33) | 1.20 (1.11 to 1.29) |
| Irregular/frequent menstrual periods | Unvaccinated | 783223773 | 27271 | 1.00 | 1.00 |
|  | 0-60 days | 11034078 | 512 | 1.18 (1.07 to 1.29) | 1.17 (1.07 to 1.29) |
|  | 61-120 days | 11024429 | 430 | 1.15 (1.04 to 1.27) | 1.15 (1.04 to 1.28) |
|  | 121-180 days | 10955653 | 392 | 1.27 (1.14 to 1.41) | 1.27 (1.14 to 1.41) |
|  | More than 180 days | 57244210 | 2345 | 1.16 (1.10 to 1.21) | 1.15 (1.10 to 1.21) |
| Intermenstrual bleeding | No infection | 814136150 | 4533 | 1.00 | 1.00 |
|  | 0-60 days | 11673689 | 62 | 0.95 (0.73 to 1.24) | 0.97 (0.74 to 1.26) |
|  | 61-120 days | 11685101 | 81 | 1.36 (1.07 to 1.73) | 1.38 (1.09 to 1.76) |
|  | 121-180 days | 11630610 | 64 | 1.29 (0.99 to 1.68) | 1.30 (1.00 to 1.69) |
|  | More than 180 days | 61070215 | 413 | 1.27 (1.13 to 1.43) | 1.28 (1.13 to 1.44) |

Adjusted for age at start of follow-up, income, education, marital status, region of birth, endometriosis, polycystic ovarian syndrome, diabetes mellitus, thyroid disorders and number of doses of vaccines against Covid-19.

**Supplementary Table S7. Confirmed Covid-19 and bleeding disturbances among women between 20 and 40 years of age restricting the follow-up to January 31^st^ 2022**

| **Outcome** | **Exposure group** | **Follow-up time in days** | **N Cases** | **Unadjusted**  **HR (95% CI)** | **Adjusted**  **HR (95% CI)** |
| --- | --- | --- | --- | --- | --- |
| Any menstrual bleeding disturbance | No infection | 597116083 | 37453 | 1.00 | 1.00 |
|  | Infected | 8275946 | 630 | 1.07 (0.99 to 1.16) | 1.03 (0.94 to 1.11) |
| Menstruation absent/scanty | No infection | 635758429 | 8137 | 1.00 | 1.00 |
|  | Infected | 9101693 | 128 | 1.06 (0.88 to 1.26) | 0.92 (0.77 to 1.11) |
| Menstruation excessive | No infection | 635483072 | 8963 | 1.00 | 1.00 |
|  | Infected | 9114672 | 175 | 1.17 (1.01 to 1.37) | 1.07 (0.91 to 1.25) |
| Irregular/frequent menstrual periods | No infection | 619422472 | 21654 | 1.00 | 1.00 |
|  | Infected | 8766031 | 384 | 1.05 (0.95 to 1.16) | 1.03 (0.93 to 1.15) |
| Intermenstrual bleeding | No infection | 641151320 | 3642 | 1.00 | 1.00 |
|  | Infected | 9238128 | 62 | 1.15 (0.89 to 1.49) | 1.07 (0.82 to 1.40) |

Adjusted for age at start of follow-up, income, education, marital status, region of birth, endometriosis, polycystic ovarian syndrome, diabetes mellitus, thyroid disorders and vaccination against Covid-19.

**Supplementary Table S8. Time since confirmed Covid-19 and bleeding disturbances among women between 20 and 40 years of age restricting the follow-up to January 31^st^ 2022**

| **Outcome** | **Exposure group** | **Follow-up time in days** | **N Cases** | **Unadjusted HR (95% CI)** | **Adjusted HR (95% CI)** |
| --- | --- | --- | --- | --- | --- |
| Any menstrual bleeding disturbance | No infection | 597116083 | 37453 | 1.00 | 1.00 |
|  | 0-60 days | 2903384 | 221 | 1.00 (0.88 to 1.15) | 0.97 (0.84 to 1.10) |
|  | 61-120 days | 1358904 | 91 | 1.00 (0.81 to 1.23) | 0.96 (0.78 to 1.18) |
|  | 121-180 days | 1076892 | 80 | 1.13 (0.90 to 1.40) | 1.06 (0.85 to 1.32) |
|  | More than 180 days | 2936766 | 238 | 1.17 (1.02 to 1.33) | 1.12 (0.98 to 1.27) |
| Menstruation absent/scanty | No infection | 635758429 | 8137 | 1.00 | 1.00 |
|  | 0-60 days | 3185536 | 43 | 1.00 (0.74 to 1.36) | 0.91 (0.67 to 1.23) |
|  | 61-120 days | 1490899 | 13 | 0.67 (0.39 to 1.15) | 0.58 (0.34 to 1.01) |
|  | 121-180 days | 1181133 | 8 | 0.53 (0.26 to 1.05) | 0.45 (0.22 to 0.90) |
|  | More than 180 days | 3244125 | 64 | 1.48 (1.15 to 1.89) | 1.28 (0.99 to 1.66) |
| Menstruation excessive | No infection | 635483072 | 8963 | 1.00 | 1.00 |
|  | 0-60 days | 3180058 | 58 | 1.04 (0.80 to 1.36) | 0.97 (0.75 to 1.27) |
|  | 61-120 days | 1493562 | 24 | 1.02 (0.69 to 1.53) | 0.93 (0.62 to 1.40) |
|  | 121-180 days | 1185652 | 31 | 1.69 (1.18 to 2.40) | 1.50 (1.05 to 2.15) |
|  | More than 180 days | 3255400 | 62 | 1.19 (0.92 to 1.53) | 1.07 (0.83 to 1.40) |
| Irregular/frequent menstrual periods | Unvaccinated | 619422472 | 21654 | 1.00 | 1.00 |
|  | 0-60 days | 3065398 | 137 | 0.98 (0.83 to 1.16) | 0.96 (0.81 to 1.14) |
|  | 61-120 days | 1438364 | 62 | 1.11 (0.87 to 1.43) | 1.09 (0.85 to 1.41) |
|  | 121-180 days | 1140903 | 50 | 1.15 (0.87 to 1.52) | 1.12 (0.85 to 1.49) |
|  | More than 180 days | 3121366 | 135 | 1.06 (0.89 to 1.26) | 1.06 (0.89 to 1.26) |
| Intermenstrual bleeding | No infection | 641151320 | 3642 | 1.00 | 1.00 |
|  | 0-60 days | 3225438 | 24 | 1.21 (0.80 to 1.83) | 1.17 (0.78 to 1.77) |
|  | 61-120 days | 1513019 | 8 | 0.91 (0.45 to 1.82) | 0.87 (0.43 to 1.75) |
|  | 121-180 days | 1200695 | 9 | 1.34 (0.69 to 2.57) | 1.23 (0.64 to 2.38) |
|  | More than 180 days | 3298976 | 21 | 1.13 (0.73 to 1.74) | 1.01 (0.65 to 1.58) |

Adjusted for age at start of follow-up, income, education, marital status, region of birth, endometriosis, polycystic ovarian syndrome, diabetes mellitus, thyroid disorders and vaccination against Covid-19.
